# Ascoidea xinghuacunensis sp. nov., a novel ascomycetous yeast species from Xinghuacun Fenjiu old workshop, Shanxi province of China

**DOI:** 10.1099/ijsem.0.006700

**Published:** 2025-03-03

**Authors:** Pan Zhen, Hai-Yan Zhu, Ying Han, Lu-Jun Luo, Liang-Chen Guo, Shuang Hu, Yu-Hua Wei, Zhang Wen, Da-Yong Han, Zhong-Wei Lin, Feng-Yan Bai, Pei-Jie Han

**Affiliations:** 1 Technology Center, Shanxi Xinghuacun Fenjiu Distilery Co. Ltd., Fenyang, Shanxi 032205, PR China; 2 State Key Laboratory of Mycology, Institute of Microbiology, Chinese Academy of Sciences, Beijing 100101, PR China; 3 College of Life Sciences, University of Chinese Academy of Sciences, Beijing 100049, PR China

**Keywords:** *Ascoidea xinghuacunensis *sp. nov., ascomycetous yeast, phylogeney, taxonomy

## Abstract

Four yeast strains belonging to the ascomycetous yeast genus *Ascoidea* were isolated from the mixture of remnants of steamed sorghum, Daqu powder and the fermented grain that fell off during transportation on the ground in the workshop which were collected in Xinghuacun Fenjiu old workshop, Shanxi province, PR China. We propose the name *Ascoidea xinghuacunensis* sp. nov. (holotype: strain CGMCC 2.7787) for the new species, which differs phenotypically from the other three species in this genus: *Ascoidea asiatica*, *Ascoidea rubescens* and *Ascoidea tarda* by its ability to grow at 37 °C. This study represents the first isolation of *Ascoidea* species within the borders of China and the initial report of the *Ascoidea* species detailing the isolation from the Baijiu brewing environment.

## Introduction

The genus *Ascoidea* (*Ascomycota*, *Saccharomycotina*, *Saccharomycetes*, *Ascoideales* and *Ascoideaceae*) was first established by Brefeld and Lindau in 1891 with *Ascoidea rubescens* as the type species [1]. This genus is remarkable within the ascomycetous yeasts for its production of asci containing an unusual number of ascospores, exceeding the typical count of 4–8 ascospores found in other members of this phylum [2,3]. Historically, seven *Ascoidea* species have been described: viz. *A. rubescens* Brefeld & Lindau [1], *Ascoidea saprolegnioides* Holtermann [4], *Ascoidea hylecoeti* Batra & Francke-Grosmann [5], *Ascoidea asiatica* Batra & Francke-Grosmann [6], *Ascoidea africana* Batra & Francke-Grosmann [6], *Ascoidea corymbosa* Gams & Grinbergs [7] and *Ascoidea tarda* Kurtzman & Robnett [3]. In the fifth edition of *The Yeasts, a Taxonomic Study* [3], de Hoog and Smith accepted three *Ascoidea* species viz. *A. africana*, *A. hylecoeti* and *A. rubescens*. Subsequently, Kurtzman and Robnett reassessed the phylogenetic relationships among *Ascoidea* species based on the phylogenetic analysis of concatenated nuclear gene sequences for the large and small subunits ribosomal DNA (LSU and SSU), translation elongation factor 1-*α* (*TEF1-α*) and the largest and second largest subunits of the RNA polymerase II (*RPB1 and RPB2*), which culminated in the proposal of the genus *Alloascoidea* to accommodate two species previously classified under *Ascoidea* [3]. In their 2013 taxonomic revision, Kurtzman and Robnett confirmed three species within *Ascoidea*, *A. asiatica*, *A. rubescens* and *A. tarda*, and two species within the newly described *Alloascoidea*, namely *Alloascoidea hylecoeti* (formerly *A. hylecoeti*) and *Alloascoidea africana* (formerly *A. africana*) [2,3]. Kurtzman and Robnett also addressed the conspecificity of *A. corymbosa*, reporting that *A. corymbosa* CBS 457.69 (type strain) and *A. africana* CBS 377.68 (now recognized as *A. asiatica*) were indeed conspecific based on D1/D2 sequence analysis in 1998 [8] and further confirmed by LSU, SSU, *TEF-1α*, *RPB1* and *RPB2* sequence analysis in 2013 [3]. Batra and Francke-Grosmann suggested in 1961 that *A. saprolegnioides* might be conspecific with *A. rubescens*, although cultures of the former are not known to exist [5]. Consequently, the genus *Ascoidea* currently comprises three distinct species. All known three *Ascoidea* species are associated with decaying wood, bark beetles and insect galleries within trees [3]. Despite its infrequent isolation, the genus *Ascoidea* is considered to have a cosmopolitan distribution, with isolates reported from Africa, Croatia, Chile and the USA; however, before this study, no strains had been reported from China [3].

During a survey of yeast diversity in Xinghuacun, a region with a long history of Baijiu (Chinese liquor) production in Shanxi province, PR China, one strain isolated from the mixture of remnants of steamed sorghum, Daqu powder, and the fermented grain that fell off during transportation on the ground in the workshop in August 2023 was found to represent a novel species in the genus *Ascoidea* based on sequence comparisons of the D1/D2 domain and the internal transcribed spacer (ITS) region. Because one strain was isolated, it is difficult to determine whether it is a rare or undersampled species. Subsequently, we sampled the same location and other similar sites, isolating three additional strains belonging to the same new *Ascoidea* species described here along with the previous strain.

## Samples collection and yeast isolation

**Fig. 1.**
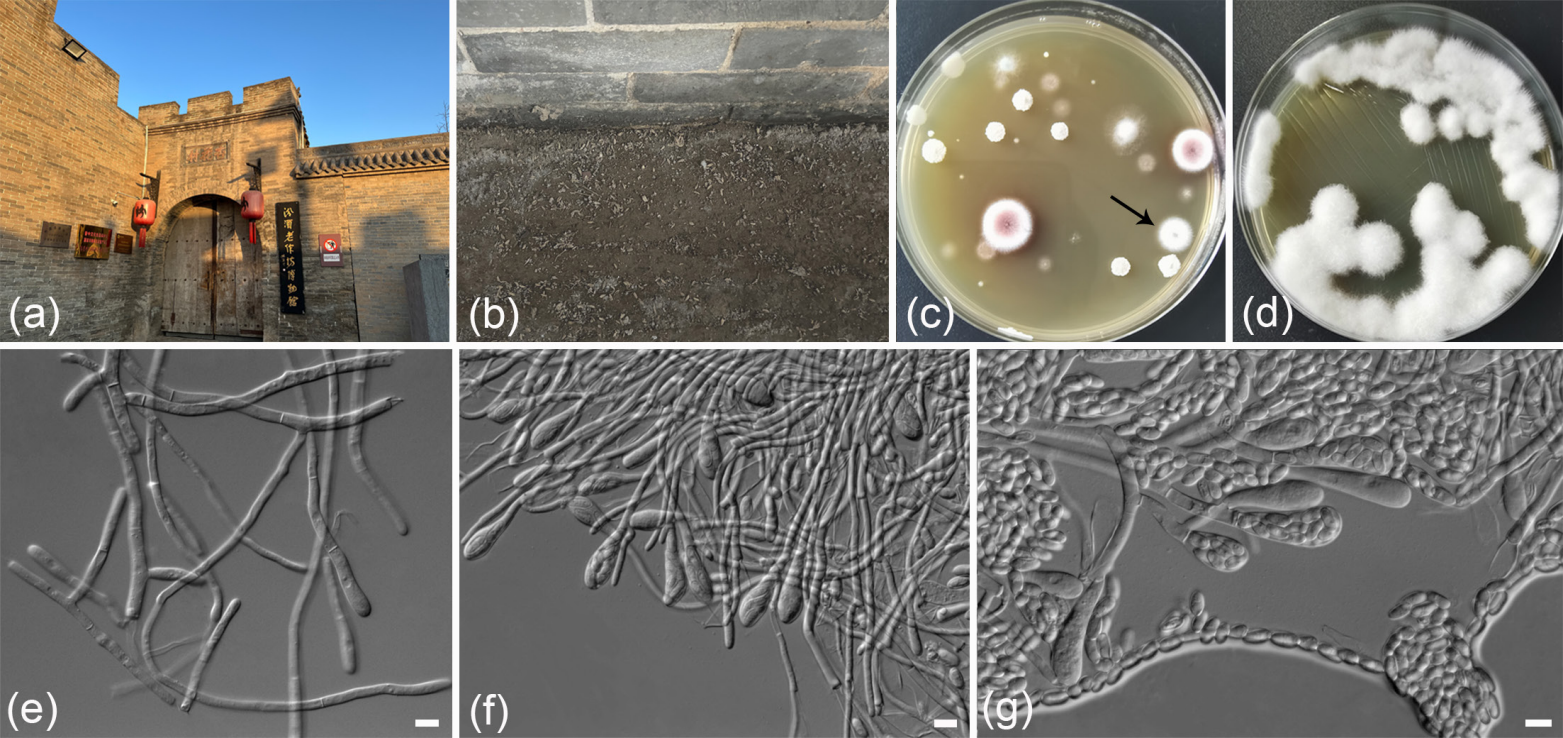
Morphology of *Ascoidea xinghuacunensis* sp. nov. (strain CGMCC 2.7787). (**a, b**) The outside and inside of the sampling workshop. (**c**) The original plate in which the CGMCC 2.7787 (indicated by arrow) was isolated. (**d**) The pure culture on YM agar after 1 week. (**e**) Septate hyphae in YM broth after 3 days. (f, g) Asci with numerous ascospores on PDA agar after 1 week. Bars, 10 µm.

**Fig. 2.**
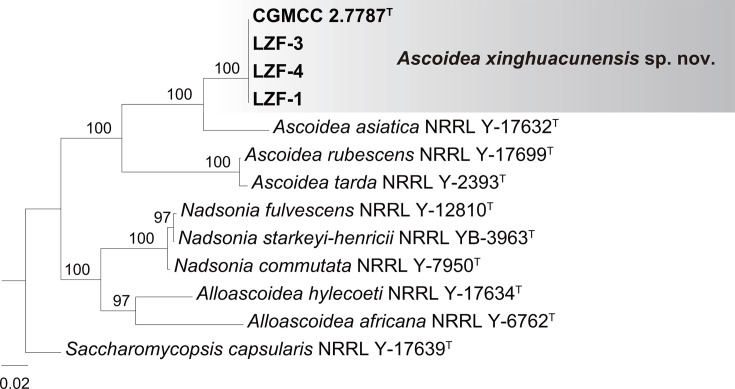
Maximum likelihood phylogenetic tree based on the concatenated D1/D2 and SSU sequences showing the phylogenetic position of *A. xinghuacunensis* sp. nov. Bootstrap percentages over 50% from 1000 replicates are shown. The species *Saccharomycopsis capsularis* is used as the outgroup. Strains marked in bold are isolated in this study. Type strains are denoted with a superscripted ‘T’. Bars, 0.02 substitutions per nucleotide position.

Samples of surface soil and remnants of grain on the ground were collected in Xinghuacun Fenjiu old workshop (approximate GPS coordinates: 37.349302° N 111.920051° E), Fenyang city, Shanxi province, PR China, in August 2023 and March 2024 (Fig. 2a, b). The samples were placed into sterile 50 ml centrifuge tubes, transported to the laboratory at room temperature and immediately subjected to yeast isolation. Approximately 5 g for each sample was suspended in 45 ml of sterile water within a 50 ml sterile centrifuge tube to create a suspension. These suspensions were then diluted to 10^−3^, 10^−4^ and 10^−5^. Aliquots of 200 µl from each dilution were plated onto yeast extract peptone dextrose agar (w/v, 2% glucose, 1% yeast extract, 2% peptone and 2% agar) supplemented with 200 µg ml^−1^ chloramphenicol. After incubation at 30 °C for 3–7 days, yeast colonies with distinct morphological characteristics were selected and purified for further analysis (Fig. 1c). The purified yeast strains (Fig. 1d) were suspended in 25% (v/v) glycerol and stored at −80 °C.

## Phenotypic characterization

Morphological characteristics and physiological and biochemical properties were examined according to standard methods described by Kurtzman *et al*. [9]. The assimilation of carbon and nitrogen compounds was conducted in liquid media. The potential sexual cycles of strains representing new species were investigated using corn meal agar (w/v, 2.5% corn starch and 2% agar), potato dextrose agar (PDA) (w/v, 20% potato infusion, 2% glucose and 2% agar), yeast extract–malt extract agar (YM) (w/v, 1% glucose, 0.3% yeast extract, 0.3% malt extract, 0.5% peptone and 2% agar) and V8 agar (w/v, 10% V8 juice and 2% agar). A loopful of cells of each test strain was inoculated separately or mixed on agar plates and incubated at 25 °C for up to 2 months and examined periodically.

## Molecular phylogenetic analysis

Genomic DNA was extracted using the method described previously [10]. The SSU of the ribosomal DNA (rDNA), the D1/D2 domain of the LSU rDNA and the ITS region were amplified and sequenced using previously described methods [11,13]. Sequence alignment was performed using MAFFT v.7 [14] and manually improved where it was necessary using mega v.7 [15]. Positions that were too ambiguous to align were excluded manually. Phylogenetic analyses based on individual D1/D2 or SSU sequences were performed based on the evolutionary distance data calculated from Kimura’s two-parameter model using the neighbour-joining algorithm executed in mega v.7 [15,17]. For the concatenated SSU and D1/D2 sequences, the maximum likelihood analyses were performed using RAxML v.8 [18]. Bootstrap analyses were performed from 1000 replicates [19].

Four yeast strains, CGMCC 2.7787, LZF-1, LZF-3 and LZF-4, were isolated from soil samples and grain remnants at the Xinghuacun Fenjiu old workshop in Shanxi province, PR China (Table 1). These strains possessed identical SSU, ITS and D1/D2 sequences, indicating they are conspecific species. When identifying these yeast strains by using blast searches through GenBank with their D1/D2 sequences as queries, no similar sequences were found.

**Table 1.** The yeast species and strains are employed in this study. The strains in bold were isolated in this study. Type strains are denoted with the superscript ‘T’

Species	Strain	Source	Origin	Accession no.		
				D1/D2	SSU	ITS
***Ascoidea xinghuacunensis*** **sp. nov.**	**JCM** **36890=CGMCC** **2.7787^T^**	**Fermented grains**	**China: Shanxi**	**PQ456465**	**PQ785825**	**PQ726416**
	**LZF-1**	**Fermented grains**	**China: Shanxi**	**PQ456462**	**PQ785822**	**PQ726413**
	**LZF-3**	**Fermented grains**	**China: Shanxi**	**PQ456463**	**PQ785823**	**PQ726414**
	**LZF-4**	**Fermented grains**	**China: Shanxi**	**PQ456464**	**PQ785824**	**PQ726415**
*A. asiatica*	CBS 377.68=NRRL Y-17632^T^	Larvae	West Africa	KC254856	KC254861	Not applicable
*A. tarda*	CBS 12609=NRRL Y-2393^T^	Flux of elm tree	USA	KC254857	KC254862	Not applicable
*A. rubescens*	CBS 116.35=NRRL Y-17699^T^	Unknown	Unknown	JQ689011	JQ698885	Not applicable
*Alloascoidea hylecoeti*	CBS 355.80=NRRL Y-17634^T^	Insect	Unknown	KC254855	KC254860	MH861273
*Alloascoidea africana*	NRRL Y-6762^T^	Unknown	Unknown	JQ689066	JQ698925	NR_159607
*Nadsonia commutata*	CBS 6640=NRRL Y-7950^T^	Field soil	East Falkland Island	KC254858	KC254863	KY104307
*Nadsonia fulvescens*	CBS 2596=NRRL Y-12810^T^	Exudate of oak tree	Russia	JQ689059	JQ698923	KY104312
*Nadsonia starkeyi-henricii*	CBS 2159=NRRL YB-3963^T^	Soil in a peat bog	Denmark	KC254859	KC254864	NR_154251
*Saccharomycopsis capsularis*	CBS 2519=NRRL Y-17639^T^	Unknown	Unknown	JQ689010	JQ698884	KY105250

The phylogenetic tree constructed from concatenated D1/D2 and SSU sequences (Fig. 2), along with two additional trees from either D1/D2 or SSU sequences alone (Fig. S1, available in the online Supplementary Material), confirmed the affinity of the new group represented by strain CGMCC 2.7787 to the genus *Ascoidea* with high bootstrap support values. The CGMCC 2.7787 group was closely related to *A. asiatica* across all three reconstructed trees (Figs 1 and S1). The CGMCC 2.7787 group exhibited 88 (15.0%, 70 substitution and 18 gaps) nucleotide differences in the D1/D2 sequences and 74 (4.9%, 63 substitution and 11 gaps) nucleotide mismatches in the SSU sequences when compared to the type strains of *A. asiatica*. These findings strongly suggest that the CGMCC 2.7787 group represents a novel species in the *Ascoidea* genus, for which the name *Ascoidea xinghuacunensis* sp. nov. is proposed.

## Phenotypical characteristics

Strains CGMCC 2.7787, LZF-1, LZF-3 and LZF-4 form cream-coloured, round, cotton-like, dry colonies with abundant hyphae and finely serrated edges, domed and matte on YM agar after growth at 25 °C for 1 week. These strains are physiologically distinct from the other three *Ascoidea* species, *A. asiatica*, *A. rubescens* and *A. tarda*, particularly in their capacity to grow on YM agar at an elevated temperature of 37 °C (Table 2).

**Table 2.** Physiological characteristics of the newly identified *A. xinghuacunensis* sp. nov. in comparison to the other three recognized *Ascoidea* species

Test	A. asiatica*	A. rubescens*	A. tarda*	*A. xinghuacunensis*
Fermentation				
Glucose	−	−	−	−
Assimilation of carbon compounds				
Glucose	+	+	+	+
Inulin	−	w	−	w
Sucrose	v	+	−	−
Raffinose	v	+	−	−
Melibiose	v	w	−	s/w
Galactose	v	w	−	−
Lactose	−	+	−	w/−
Trehalose	+/w	+	−	w
Maltose	v	+	−	w
Melezitose	+/w	w	−	s
*α*-Methyl-d-glucoside	v	w	−	−
Starch soluble	+	+	−	w
Cellobiose	+/w	+	−	w
Salicin	+/w	−	−	−
l-Sorbose	+/w	−	−	−
l-Rhamnose	v	+	−	−
d-Xylose	+	+	−	+
l-Arabinose	v	−	−	−
d-Arabinose	−	+	−	−
d-Ribose	−	+	−	−
Methanol	w	−	−	−
Ethanol	+	+	+	+
Glycerol	+	w	−	+
Erythritol	−	+	−	−
Ribitol	v	+	−	−
Galactitol	na	na	−	−
d-Mannitol	v	+	−	−
d-Glucitol	+	w	−	−
Inositol	−	w	−	−
dl-Lactate	−	−	w	+
Succinate	v	−	−	+
Citrate	v	−	−	−
d-Gluconate	−	−	−	−
d-Glucosamine	w	−	−	s/w
*N*-Acetyl-d-glucosamine	na	na	−	+
Hexadecane	na	na	−	−
Assimilation of nitrogen compounds				
Nitrate	v	−	na	+
Additional growth tests and other characteristics				
10% NaCl plus 5% glucose	na	na	−	−
Starch formation	−	−	na	−
Growth at 22 °C	+	+	+	+
Growth at 25 °C	+	+	−	+
Growth at 30 °C	−	+	−	+
Growth at 37 °C	−	−	−	+
Growth at 42 °C	−	−	−	−

na, Not available; s, slow positive; v, variable; w, weakly positive; +, positive; −, negative.

* Data are from [3].

All four *Ascoidea* species, including the newly identified *A. xinghuacunensis* sp. nov. in this study and the other three recognized *Ascoidea* species, are capable of assimilating glucose and ethanol as their sole carbon sources. The four *Ascoidea* species show different heat tolerance abilities. *A. xinghuacunensis* sp. nov. can grow at 37 °C, which is the most heat-tolerant species in the genus *Ascoidea. A. rubescens* possesses the ability to grow at 30 °C but not at 37 °C, whereas *A. asiatica* can grow at 25 °C, but not at 30 °C. Furthermore, *A. tarda* exhibits the capability to grow at a lower temperature of 22 °C, but not at 25 °C (Table 2).

## Description of *Ascoidea xinghuacunensis* sp. nov. H.Y. Zhu, L.J. Luo and P.J. Han

*Ascoidea xinghuacunensis* (xing.hua.cun.en’sis. N.L. fem. adj. *xinghuacunensis*, pertaining to Xinghuacun, referring to the location for which the species was isolated).

Culture characteristics: After cultivation in YM broth at 25 °C for 3 days, the slender, branched and septate true hyphae measured 2.3–4.9 µm in diameter (Fig. 1e). Following a month-long growth in YM broth at the same temperature, cotton-like mycelium was observed suspended in the liquid medium, with no sedimentation or pellicle formation. After growth on YM agar for 1 week at 25 °C, cultures were cream-coloured, round, cotton-like, dry, with abundant aerial mycelium and finely serrated edges, domed and matte (Fig. 1d). Asci with more than four ascospores were observed on PDA and V8 agar after 1 week at 25 °C (Fig. 2f, g). Asci are single, formed terminally on hyphae or lateral branchlets and are clavate, 4.8–14.9×45.8–67.6 µm. Ascospores are ellipsoidal, 3.8–4.1×8.2–8.9 µm. The species is homothallic.

Physiological and biochemical characteristics: Glucose is not fermented. Glucose, maltose (weak), cellobiose (weak), trehalose (weak), lactose (weak/negative), melibiose (slow/weak), melezitose (slow), inulin (weak), starch soluble (weak), d-xylose, d-glucosamine (slow/weak), ethanol, glycerol, dl-lactic acid, succinic acid and *N*-acetyl-d-glucosamine are assimilated as sole carbon sources. d-Galactose, l-sorbose, sucrose, raffinose, l-arabinose, d-arabinose, d-ribose, l-rhamnose, methanol, erythritol, ribitol, galactitol, d-mannitol, d-glucitol, *α*-methyl-d-glucoside, salicin, d-glucuronic acid, inositol, citric acid, hexadecane and xylitol are not assimilated as sole carbon sources. Ethylamine hydrochloride, potassium nitrate, sodium nitrite, cadaverine dihydrochloride, l-lysine and ammonium sulphate are assimilated as sole nitrogen sources. Diazonium blue B reaction is negative. Extracellular amyloid compounds are not produced. Growth in 10% (w/v) sodium chloride plus 5% (w/v) glucose medium is negative. Growth on 50% (w/v) glucose–yeast extract agar and 60% (w/v) glucose–yeast extract agar is negative. Growth in urease agar is negative. Growth in vitamin-free medium is positive. Growth occurs on YM agar at 37 °C, but not at 40 °C.

The holotype, CGMCC 2.7787, was isolated from the samples of the mixture of remnants of steamed sorghum, Daqu powder and the fermented grain that fell off during transportation on the ground in the workshop which were collected in Xinghuacun Fenjiu old workshop, Fenyang city, Shanxi province, PR China, in August 2023 and has been deposited in a metabolically inactive state in the China General Microbiological Culture Collection Center (CGMCC), Beijing, China. The ex-type culture has been deposited in the Japan Collection of Microorganisms (JCM), Koyadai, Japan, as JCM 36890. The GenBank/EMBL/DDBJ accession numbers for the LSU D1/D2, ITS and SSU sequences of the strain CGMCC 2.7787 are PQ456465, PQ726416 and PQ785825; of strain LZF-1 are PQ456462, PQ726413 and PQ785822; of strain LZF-3 are PQ456463 PQ726414 and PQ785823; and of strain LZF-4 are PQ456464, PQ726415 and PQ785824, respectively. The Fungal Names number of *A. xinghuacunensis* sp. nov. is FN572126.

## supplementary material

10.1099/ijsem.0.006700 Uncited Fig. S1.

## Abbreviations

CGMCC: China General Microbiological Culture Collection Center
ITS: internal transcribed spacer
JCM: Japan Collection of Microorganisms
LSU: large subunit
PDA: potato dextrose agar
rDNA: ribosomal DNA
*RPB1*: the largest subunit of the RNA polymerase II
*RPB2*: the second largest subunit of the RNA polymerase II
SSU: small subunit
*TEF1*-*α*: translation elongation factor 1-*α*
YM: yeast extract–malt extract

## References

[1] Brefeld O, Lindau G (1891). Die formen der Hemiasci und ihre Kultur in Nährlösungen. Unters Gesammtgeb Mykol.

[2] de Hoog GS, Smith MTH, Kurtzman CP, Fell JW, Boekhout T (2011). The Yeasts, A Taxonomic Study.

[3] Kurtzman CP, Robnett CJ (2013). *Alloascoidea hylecoeti* gen. nov., comb. nov., *Alloascoidea africana* comb. nov., *Ascoidea tarda* sp. nov., and *Nadsonia starkeyi-henricii* comb. nov., new members of the *Saccharomycotina* (*Ascomycota*). FEMS Yeast Res.

[4] Holtermann C (1898). Mykologische Untersuchungen aus den Tropen.

[5] Batra LR, Francke‐Grosmann H (1961). Contributions to our knowledge of ambrosia fungi. I. *Ascoidea hylecoeti* sp. nov. (*Ascomycetes*). Am J Bot.

[6] Batra LR, Francke-Grosmann H (1964). Two new ambrosia fungi – *Ascoidea asiatica* and *A. africana*. Mycologia.

[7] Gams W, Grinbergs J (1970). *Ascoidea corymbosa* n. spec., ein hefëahnlicher Pilz im Bast von *Araucaria araucana*. Acta Bot Neerl.

[8] Kurtzman CP, Robnett CJ (1998). Identification and phylogeny of ascomycetous yeasts from analysis of nuclear large subunit (26S) ribosomal DNA partial sequences. Antonie Van Leeuwenhoek.

[9] Kurtzman CP, Fell JW, Boekhout T, Robert V, Kurtzman CP, Fell JW, Boekhout T (2011). The Yeasts, A Taxonomic Study.

[10] Zhu HY, Wei YH, Guo LC, Wen Z, Hu S (2025). Two new arthroconidial yeast species from bark and pit mud in China. MycoKeys.

[11] Wang QM, Bai FY (2004). Four new yeast species of the genus *Sporobolomyces* from plant leaves. FEMS Yeast Res.

[12] Sugita T, Nakase T (1999). Non-universal usage of the leucine CUG codon and the molecular phylogeny of the genus *Candida*. Syst Appl Microbiol.

[13] Wang QM, Bai FY, Zhao JH, Jia JH (2003). *Bensingtonia changbaiensis* sp. nov. and *Bensingtonia sorbi* sp. nov., novel ballistoconidium-forming yeast species from plant leaves. Int J Syst Evol Microbiol.

[14] Katoh K, Standley DM (2013). MAFFT multiple sequence alignment software version 7: improvements in performance and usability. Mol Biol Evol.

[15] Kumar S, Stecher G, Tamura K (2016). MEGA7: molecular evolutionary genetics analysis version 7.0 for bigger datasets. Mol Biol Evol.

[16] Lachance MA (2022). Phylogenies in yeast species descriptions: in defense of neighbor-joining. Yeast.

[17] Kimura M (1980). A simple method for estimating evolutionary rates of base substitutions through comparative studies of nucleotide sequences. J Mol Evol.

[18] Stamatakis A (2014). RAxML version 8: a tool for phylogenetic analysis and post-analysis of large phylogenies. Bioinformatics.

[19] Felsenstein J (1985). Confidence limits on phylogenies: an approach using the bootstrap. Evolution.

