# Supplementary material for: Ascoidea xinghuacunensis sp. nov., a novel ascomycetous yeast species from Xinghuacun Fenjiu old workshop, Shanxi province of China
Source: Int J Syst Evol Microbiol. 2025 Mar 3;75(3):006700. doi: 10.1099/ijsem.0.006700 (PMC11876787; doi:10.1099/ijsem.0.006700)
Supplement: Uncited Fig. S1. [file ijsem-75-06700-s001.pdf]

Supplementary materials

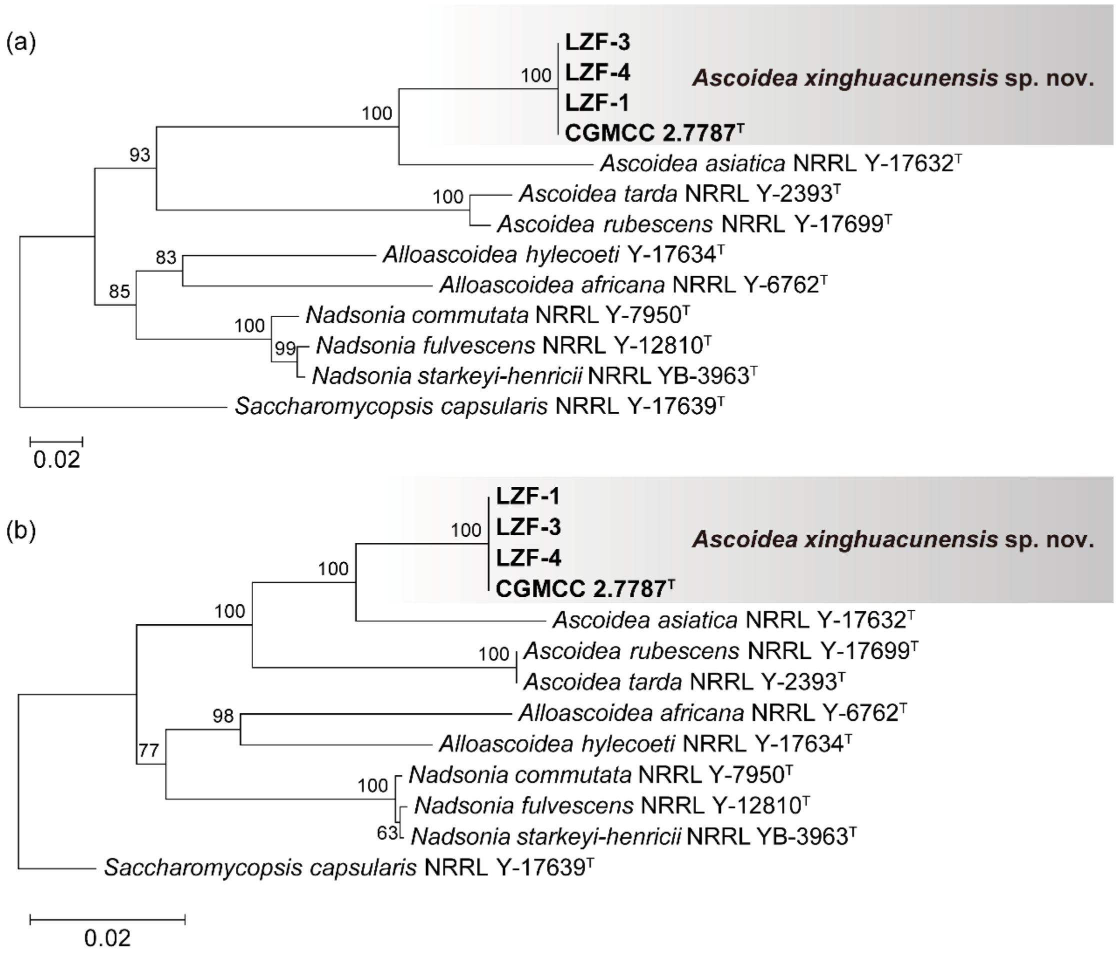

Fig. S1 Neighbor-Joining phylogenetic tree based on the D1/D2 sequences (a) and the SSU sequences (b) showing the phylogenetic position of *Ascoidea xinghuacunensis* sp. nov. Bootstrap percentages over 50% from 1000 replicates are shown. The species *Saccharomycopsis capsularis* is used as the outgroup. Strains marked in bold were isolated in this study. Type strains are denoted with a superscripted 'T'. Bars, 0.02 substitutions per nucleotide position.
